# Effect of Initial Crack Position on Crack Propagation Behaviors of Heavy-Duty Transmission Gear

**DOI:** 10.3390/ma16175961

**Published:** 2023-08-31

**Authors:** Yingtao Zhang, Zirong Tang, Lijuan Zhao, Benxiang Gong, Gang Wang, Zhichao Li

**Affiliations:** 1 College of Mechanical & Electrical Engineering, Hohai University, Changzhou 213022, China; tangzirong@hhu.edu.cn (Z.T.); 19981085@hhu.edu.cn (L.Z.); gongbenxiang@foxmail.com (B.G.); 2 Beijing Key Lab of Precision/Ultra-Precision Manufacturing Equipments and Control, Tsinghua University, Beijing 100084, China; gwang@tsinghua.edu.cn; 3 DANTE Solutions, Inc., Cleveland, OH 44130, USA; charlie.li@dante-solutions.com

**Keywords:** initial crack, crack propagation, numerical simulation, XFEM

## Abstract

The tooth bending fatigue fracture is caused by the alternating loads for the heavy-duty transmission gears. The crack initiation and propagation are the two major parts in the failure process. The crack propagation behavior is mainly affected by initial crack position except for the load and material properties. In this paper, the crack propagation model of a gear is established under the considering of crack initiation location by using extended finite element method (XFEM). The model accuracy is verified by testing results of strain and fractography by conducting the single-tooth bending fatigue experiment. The influence of crack initiation locations on subsequent crack propagation behavior is analyzed. The crack length in the tooth width direction and depth direction is faster when the initial crack is located in the middle of root surface. The crack growth rate is lower for the initial crack located in the surface close to the end surface of the gear.

## 1. Introduction

Heavy-duty transmission gear was a crucial component in the mechanical equipment’s transmission system [1]. The fatigue failure leading to fracture took place at the tooth root when subjected to cyclic loads [2]. The fracture process of gear teeth generally included two processes of crack inception and crack advancement [3]. When a minute fissure emerges on the surface of the gear at the root of the tooth, it propagates towards the peak principal stress direction, ultimately resulting in the fracture of the tooth [4].

A cohesive interface model using molecular dynamics simulations was developed to explore the impact of diverse cyclic loading conditions on fatigue crack propagation [5]. ANSYS and FRANC3D software were utilized to calculate the stress intensity factors and forecast the trajectory of fatigue crack growth [6]. An improved gear dynamics model was established by using the slice method, which provided theoretical reference for the diagnosis of gear tooth root crack [7]. For spiral bevel gears with more complex structures, three-dimensional fatigue crack trajectories under non-proportional loads were analyzed using boundary element analysis and linear elastic fracture mechanics principles [8]. When these finite element methods were employed to model crack growth, the fine mesh was required to be applied around the crack tip and the modeling steps were complex, resulting in low efficiency of the simulation process [9].

The XFEM was widely used in the simulation of crack propagation without refining the mesh at the crack tip [10]. By extending the shape function to articulate the discontinuous displacement field, the crack was independent of the mesh [11], which was efficient to be applied for the crack with complex shape [12]. The influence of initial crack geometry and material parameters was studied by XFEM on the fatigue life. The fatigue life is primarily influenced by the material coefficient C and the ultimate tensile strength [13]. Researchers utilized the extended finite element method (XFEM) to study the variation in mesh rigidity over time (TVMS) and the characteristics of fracture propagation in spur gears. When the backup ratio exceeds 0.3, cracks extend towards the tooth thickness, whereas a backup ratio equal to or less than 0.3 results in cracks propagating towards the gear rim [14]. The behavior of crack propagation was primarily influenced by the applied load, material characteristics, and the initial size of the crack. Additionally, in real-world operating conditions, the location of the initial crack also played a significant role in determining the crack propagation behavior [15]. The previous studies of fatigue life and failure form were focused on the S-N model and failure analysis. Also, relatively little attention was paid to the specific failure evolution law in the actual gear application.

In this paper, the crack propagation model of a spur gear was established with the considering of crack initiation position by using XFEM in ABAQUS. The model accuracy was verified by testing results of strain and fractography by conducting the single-tooth bending fatigue experiment. The simulation models with initial crack located on different positions were built to study the effects of initial crack position on the crack propagation law and speed under the working conditions. The key position can be determined when the propagation life is lowest or longest. The life of crack initiation is related to the material and surface strengthening process, so the range of fatigue life can be accurately calculated by considering the propagation process with different initial crack positions.

## 2. Modeling and Experimental Methods

### 2.1. Numerical Model

The displacement fields were described with standard finite element methods by using shape function and element mesh nodes:

(1) $$u_{fem}(x)=\sum_{i\in S}N_{i}(x)u_{i}$$

where *S* denotes the complete set of nodes in the mesh, *N_i_* (*x*) represents the shape functions, and *u_i_* represents the nodal displacements of the elements.

For the XFEM, the displacement field description of the two-dimensional crack model can be written in the general form as [16]:

(2) $$u_{xfem}(x)=\sum_{i=1}^{n}N_{i}(x)u_{i}+\sum_{i\in K_{b}}N_{i}(x)P(x)a_{i}+\sum_{i\in K_{s}}\sum_{j=1}^{4}N_{i}(x)\gamma_{j}(x)a_{i}b_{ij}$$

where *N_i_* (*x*) are the shape functions. *n* is the global nodes number [17]. *K_b_* or *K_s_* denotes the nodes of an element that is entirely or partially intersected by the crack respectively. *a_i_* represents the enriched nodal degrees of freedom related to the Heaviside function *P*(*x*). *b_ij_* corresponds to the enriched nodal degrees of freedom vector values related to the crack-tip enrichment function *γ_j_*(*x*) [13]. XFEM is a new method developed on the basis of standard finite method, which is more suitable for complex crack propagation simulation [18].

In low-cycle fatigue analysis, when a part was loaded between its maximum and minimum loads, the initiation criterion of fatigue crack growth is represented by the relative fracture energy release rate Δ*G*. Fatigue crack growth initiation criterion in ABAQUS is defined as [19]:

(3) $$f=\frac{N}{C_{1}{\left(\Delta G\right)}^{C_{2}}}\geq 1.0$$

where *C*_1_ and *C*_2_ represent material coefficients, while *N* signifies the number of cycles. Unless the above equation is satisfied, the interface elements located at the crack tips will remain unreleased.

The fatigue crack growth can be described by using the Paris’ law which relates sub-critical crack growth rate (*da*/*dN*) to the range of the fracture energy release rate (Δ*G*) during the fatigue cycle as:

(4) $$\frac{da}{dN}=C_{3}{\left(\Delta G\right)}^{C_{4}}$$

where the values of the material constants *C*_3_ and *C*_4_ can be determined through three-point bending experiments.

**Figure 1:**
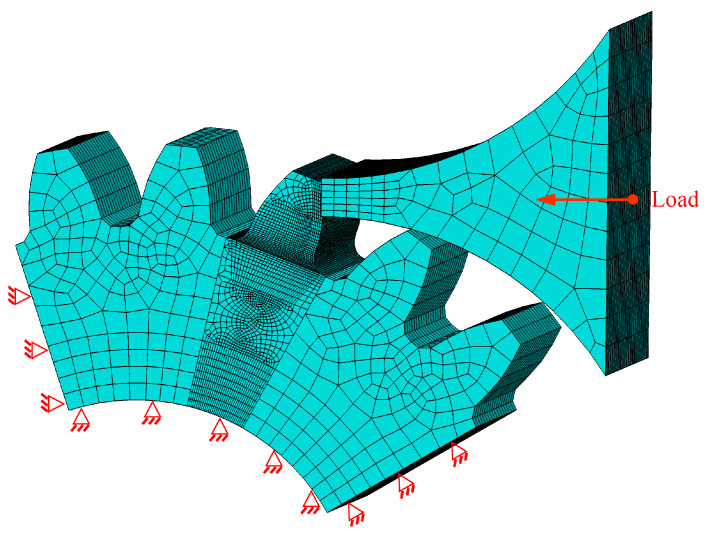
Single-tooth bending model.

The single-tooth bending 3D stress model was established by using the finite element software ABAQUS without crack propagation. Table 1 presents the parameters for the gear and material. A cyclic load, perpendicular to the tooth profile, was applied on the pressure head with *F*_min_ = 1.6 kN and *F*_max_ = 40 kN. With the minimum size 0.2 mm and maximum size 2 mm, the finite element mesh consisted of 102,840 nodes and 95,207 elements, and it was accompanied by the boundary conditions illustrated in Figure 1.

**Figure 2:**
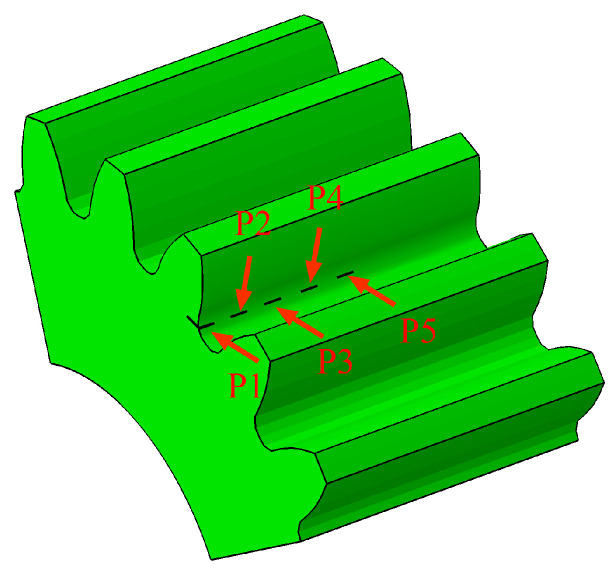
The location of the initial crack setting.

Based on the 3D stress model and the material parameters, the crack expand model with single initial inception was built to study the process of the crack expand and the stress change. The initial crack with five positions was set in the tooth root surface respectively and is illustrated in Figure 2. The crack was defined as rectangle with length 1 mm and width 0.5 mm. The distance between the initial crack at different positions and the end face of the gear tooth is depicted in Table 2.

### 2.2. The Gear Material and Heat Treatment Process

**Figure 3:**
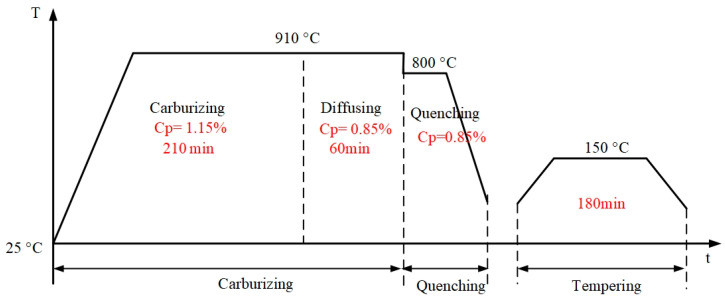
Carburizing and quenching process curve.

The 20Cr2Ni4A steel was used in the manufacturing of the experimental gear, and the chemical composition is shown in Table 3. After the tooth cutting process was finished, the carburizing and quenching process was applied to improve the strength. The whole heat treatment process consisted of carburizing, quenching, and tempering process; the process curve is shown in Figure 3.

**Figure 4:**
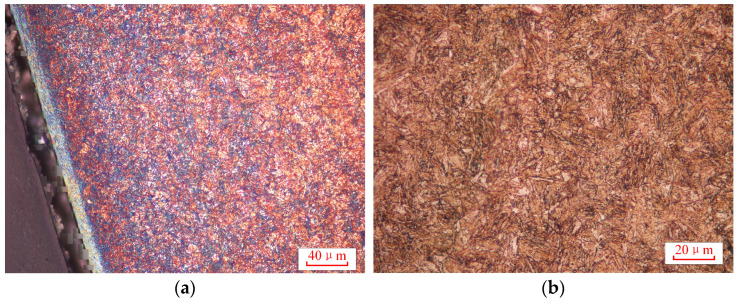
Metallographic microstructure: (**a**) interface at surface; (**b**) core.

After heat treatment process, the microstructures were observed by optical microscope. It was mainly martensite on the surface, and the mixture of martensite and austenite was in the carburizing layer, as shown in Figure 4a. It was mainly bainite in the core of the tooth, as shown in Figure 4b.

### 2.3. Single-Tooth Bending Fatigue Testing

**Figure 5:**
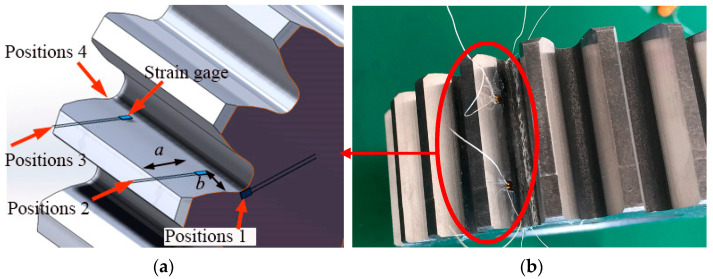
Strain gauges configuration: (**a**) location diagram; (**b**) experimental gear.

**Figure 6:**
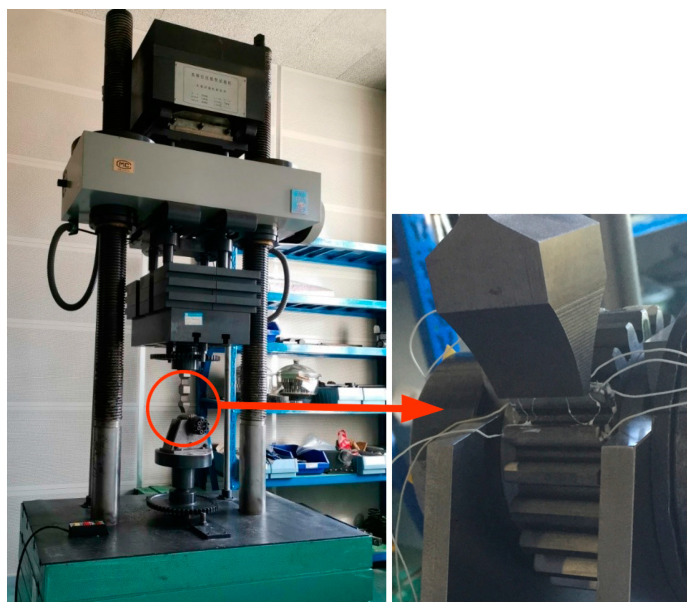
Images of the single-tooth bending specimen loading.

The gear bending fatigue experiment was performed as a validation for the simulation model’s accuracy. During the testing procedure, strain history at various positions on the gear was captured using a strain gauge and a Graphtec GL7000 data recorder, which operated at a sampling rate of 1000 Hz. The strain gauge sticking was assembled on gear surface at different four positions, as shown in Figure 5a, and the experimental gear for fatigue testing is shown in Figure 5b. The position coordinates are listed in Table 4 for the strain gauge located on the gear surface. Position 1 and Position 4 were located on the end face close to the root. After connecting the strain gauge with the data logger, transfer the gear into the PLG-300C high-frequency testing machine (as illustrated in Figure 6). Table 5 presents the load parameters and the corresponding root bending stress values.

## 3. Results and Discussions

### 3.1. Simulation Results

**Figure 7:**
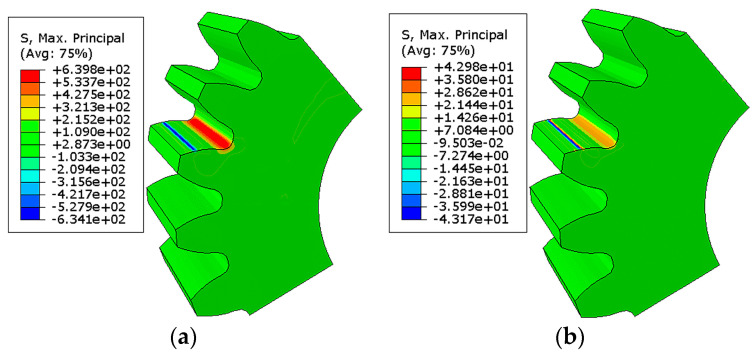
Results of root stress during cyclic loading: (**a**) root stress when cyclic load reached the maximum value; (**b**) root stress when cyclic load reached the minimum value.

The stress was analyzed along with the load without initial crack. In the cyclic loading process, the maximum bending stress was about 639 MPa (strain value: 0.0031) on the root surface, as illustrated in Figure 7a. Also, the minimum stress was about 43 MPa, as illustrated in Figure 7b. The maximum stress appeared on the root of the tooth and was consistent with calculation results of theoretical formula.

**Figure 8:**
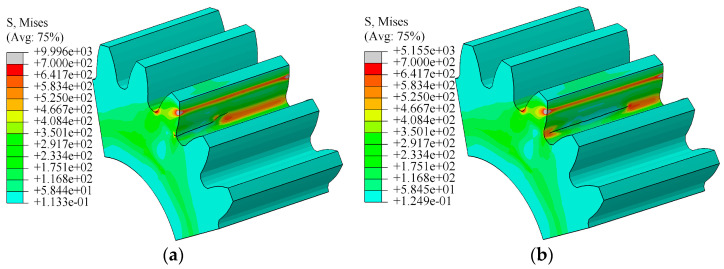
Crack propagation process of initial crack at different positions: (**a**) the initial crack was located at position P3; (**b**) the initial crack was situated at position P4.

After about 60,000 cycles, the crack size was affected by the position of initial crack, as illustrated in Figure 8a,b. When the initial fissure was situated at position P4, the length was longer than that at position P4 in the direction of tooth width.

### 3.2. Testing Results of the Strain on the Root Surface

**Figure 9:**
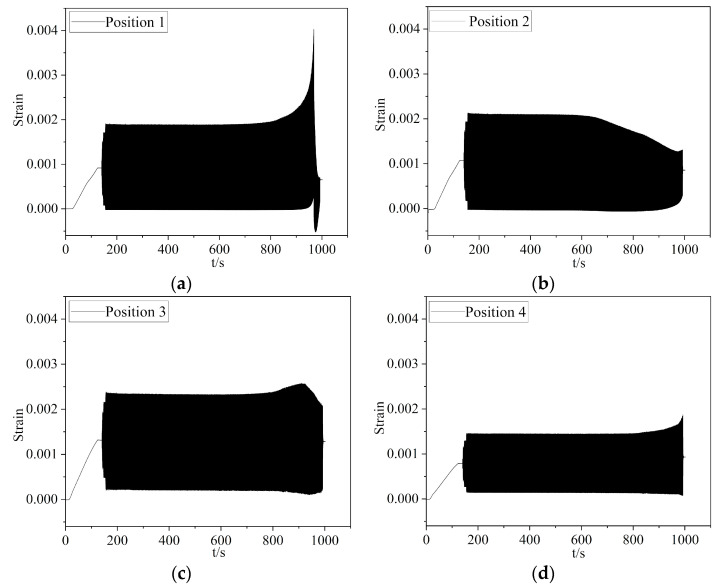
The testing results of strain at different positions: (**a**–**d**) were the strain histories of positions 1–4, respectively.

The experimental process involved capturing the strain variations, as depicted in Figure 9. Upon magnification, the strain curve exhibited a sinusoidal pattern, as depicted in Figure 9d. For the monitored location on the tooth surface, there was no noticeable strain variation before the crack occurred on root surface. However, at a certain point along the crack growth path, the stress increased as the crack tip approached that point and decreased as the crack tip moved away from it [20,21], as illustrated in Figure 9c. For the initial crack formed near the monitored position, the stress/strain was decreasing during the crack extension process, as illustrated in Figure 9b.

### 3.3. Validation of the Model

**Figure 10:**
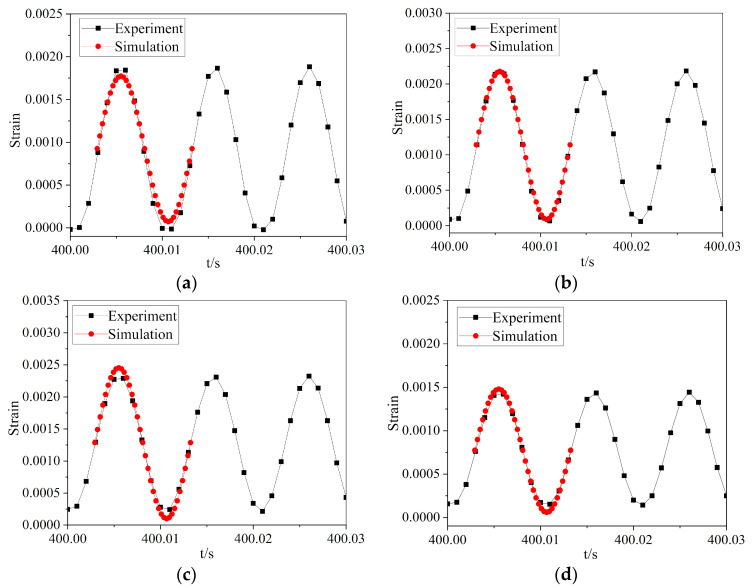
The simulation results and test results of cyclic loading curves at different positions: (**a**–**d**) are the comparisons of positions 1–4.

For the crack initiation stage, the data at about 400 s test time were selected to compare with the simulation results of the model without initial crack in one cycle. According to the coordinate of the test position, the strain data of the corresponding node of the model were output. The comparison of strain results with cyclic loading at different positions, as illustrated in Figure 10, with a maximum error of 6.4%. The simulation and test strain curves were sinusoidal, and the stress ratio was 0.04.

**Figure 11:**
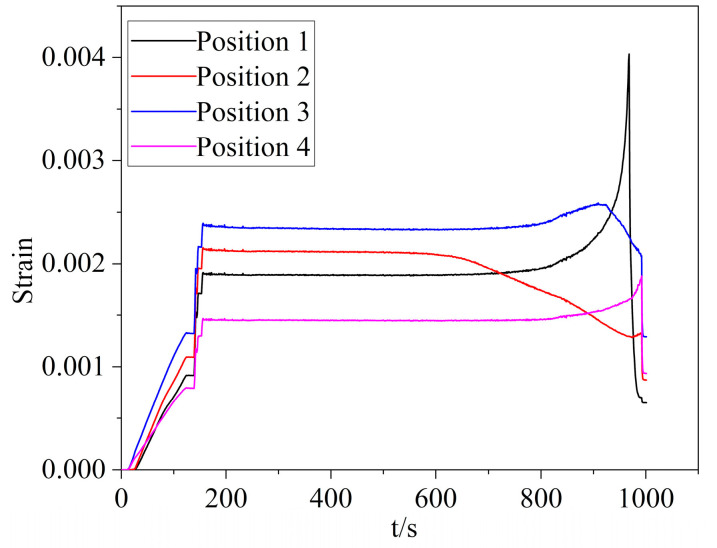
Maximum strain of each cycle at different positions.

The highest strain values at each position were selected to analyze the gear bending fatigue failure process, as illustrated in Figure 11. When the maximum value of strain changed during the fatigue process, the crack appeared near the monitored position. The strain at this position decreased as the crack appeared on the surface close to the monitor point [22].

**Figure 12:**
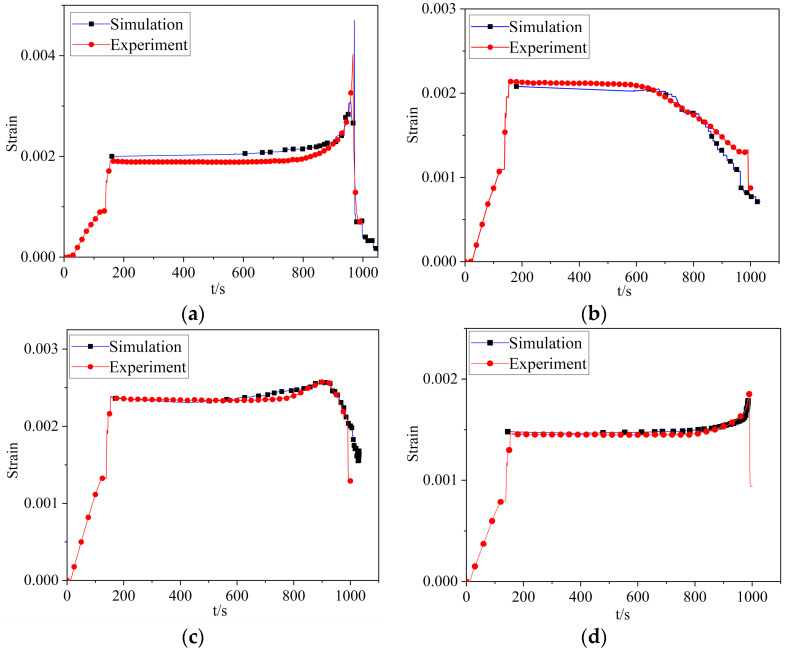
Comparison between simulation results and test results of maximum strain at different positions: (**a**–**d**) were the comparisons of positions 1–4.

According to testing results of strain, the fatigue crack initiated below position 2, which was consistent with initial crack position P3 in the simulation model. The strain at test position 2 was gradually decreasing as the crack propagated, and there was a large stress area in the front of the crack. As the crack was approaching the test point, the test strain value was increasing. As the crack was moving away from the test point, the test result was dropping. The closer the crack was to the test position, the greater the range of change, as shown in Figure 12. The simulation results of this position were selected for comparison with the test results. The correlation between the simulate and experimental results of maximum strain values at different monitored positions, with a maximum error of 15.5% at position 2. Also, the comparison results were highly consistent.

**Figure 13:**
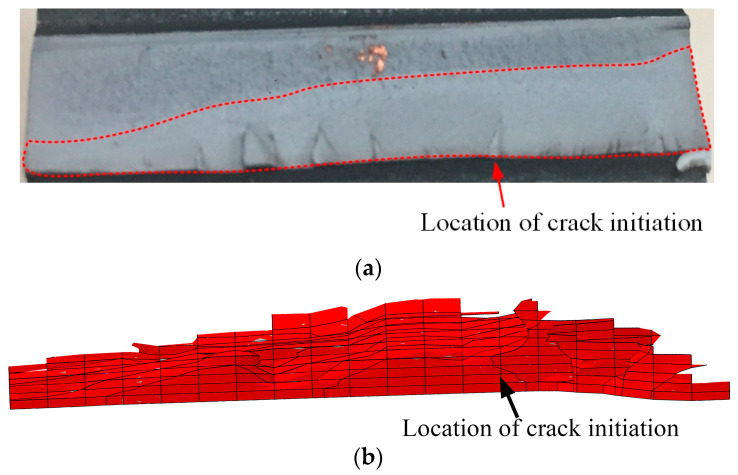
Experimental and simulated fracture surface morphology: (**a**) the experimental results; (**b**) the simulation results.

**Figure 14:**
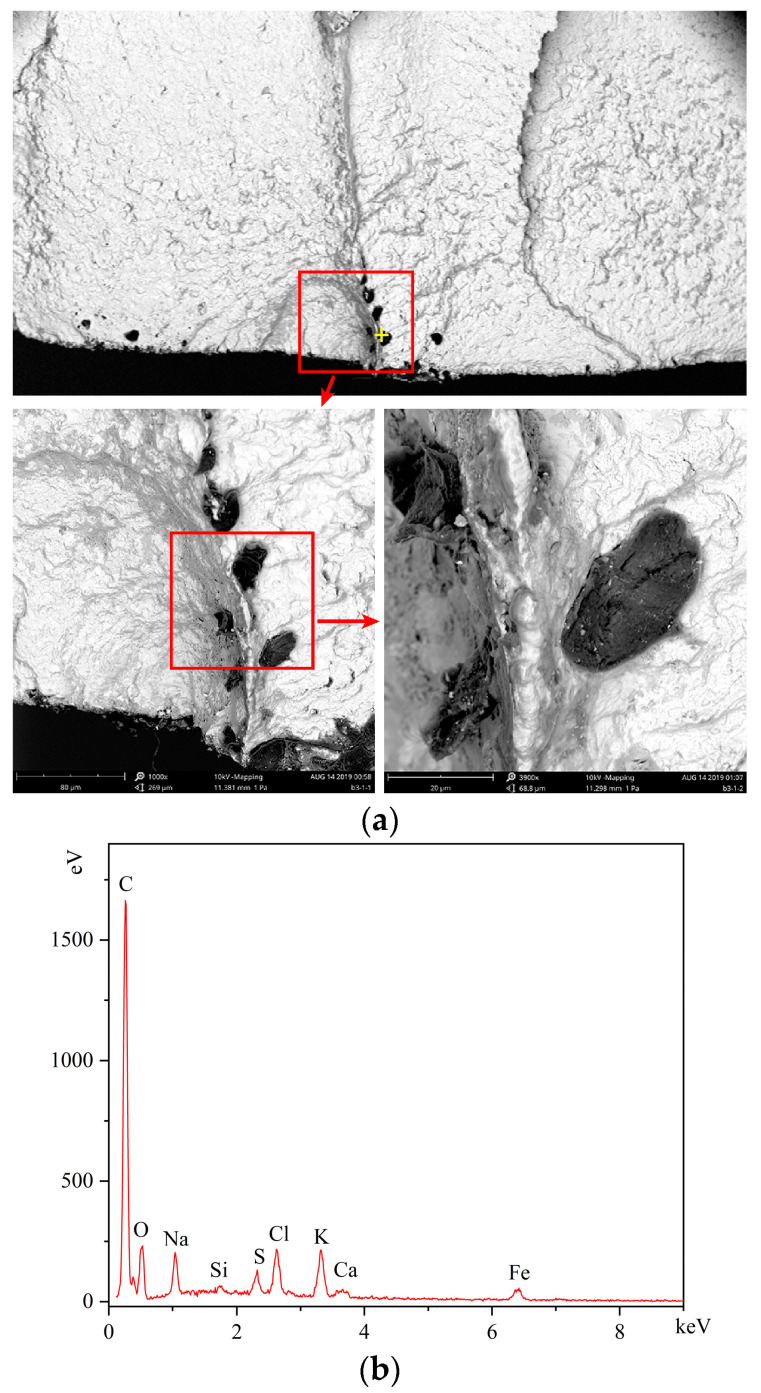
Fatigue fracture morphology of the gear: (**a**) the crack initiation area; (**b**) energy spectrum of particle.

The fracture morphology of the experimental gear is illustrated in Figure 13a. The situated of crack initiation was about one quarter of the tooth root, which was consistent with the simulation model with the crack initiation P3, as illustrated in Figure 13b. The fracture morphology of the simulation was consistent with the observations of fractured surface. SEM/EDX was used to observe the fracture surface of the crack initiation area. In the crack initiation region, i.e., the red circle, several black particles were distributed, as illustrated in Figure 14a, which was the enlarged view of the area indicated by the arrow in Figure 13. The particle was embedded in the surface material, leading to local stress concentration and fatigue crack initiation [23]. The particle was included with high carbon content based on the EDX testing results, as illustrated in Figure 14b. Inclusions were produced during steel smelting and remained in the steel [24].

Under the condition of uniform pressure head load applied to the tooth top, the initial crack appeared around the inclusion location at the corresponding 30° angle of the tooth root. It was relatively brittle for the hardened layer on tooth surface, so the crack spread fast on the surface in the width direction during the subsequent crack propagation process. The microstructure was bainite in the core, and it has better toughness. So, the crack expands slowly in the depth direction. The bending fatigue failure of the gear teeth was not a process of uniformly extending inward from the surface of the tooth root.

### 3.4. Effect of Initial Crack Location on Crack Propagation Behavior

**Figure 15:**
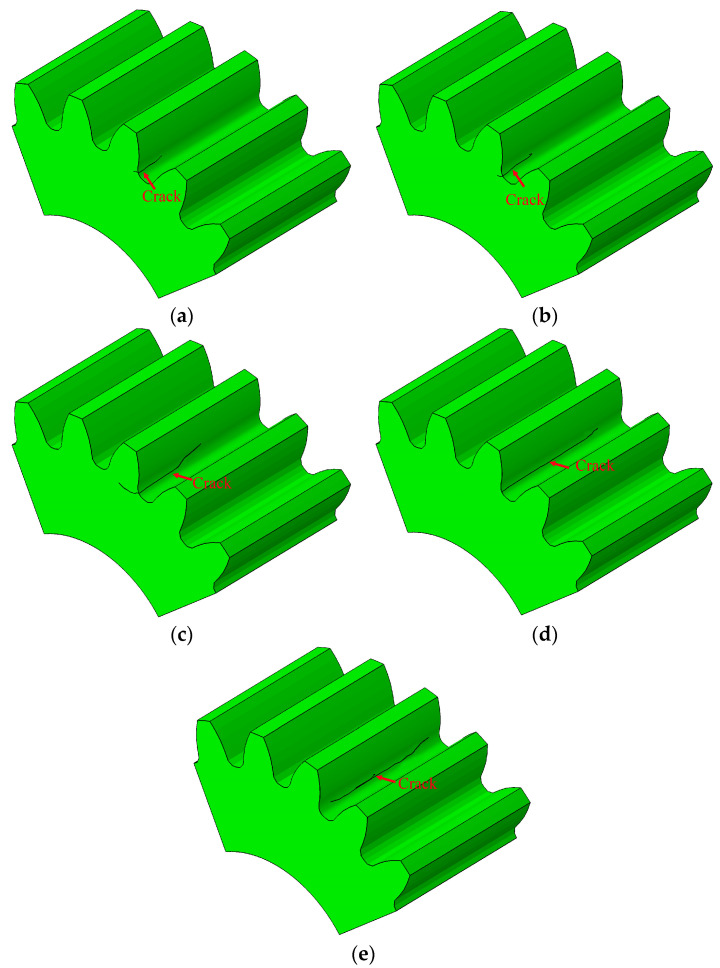
Crack propagation path in the tooth width direction: (**a**–**e**) were the simulation results of initial cracks located at positions P1–P5, respectively.

After about 70,000 cycles, the crack propagation paths in the gear were different for cracks initiated at positions P1–P5, as illustrated in Figure 15a–e. The crack in the tooth width direction was largest when the initial crack was situated in the middle of root surface.

**Figure 16:**
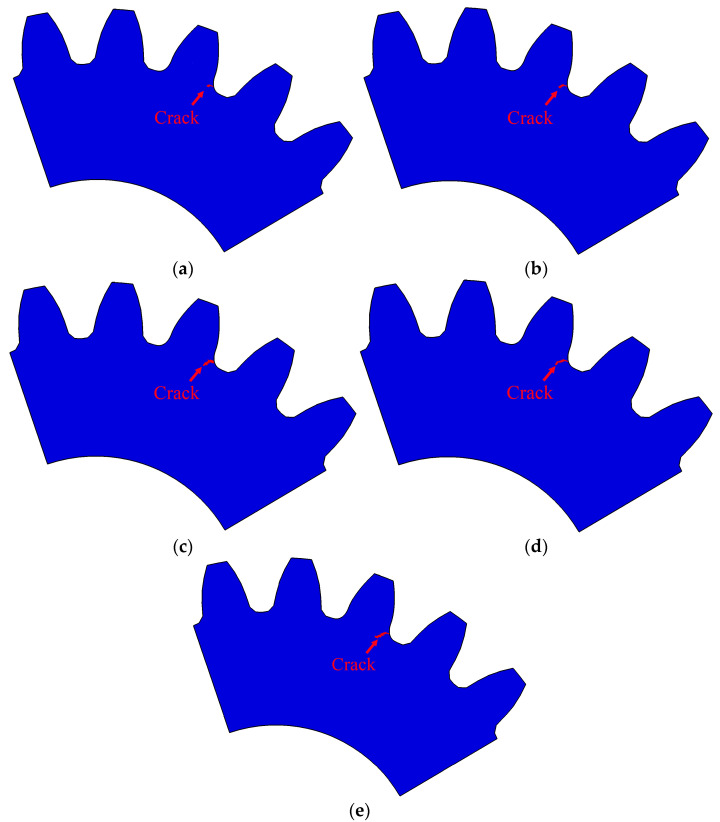
Crack growth path in the depth direction: (**a**–**e**) were the simulation results of initial cracks located at positions P1–P5, respectively.

The crack growth path in the depth direction can be obtained by outputting STATUXFEM sectional view, as illustrated in Figure 16a–e. The path in the figure was selected from the position where the crack with the maximum depth was, and the distance from the end face is illustrated in Table 6. By contrast, the depth of crack propagation was largest when the initial crack was located about one quarter of the root surface, i.e., position P3.

**Figure 17:**
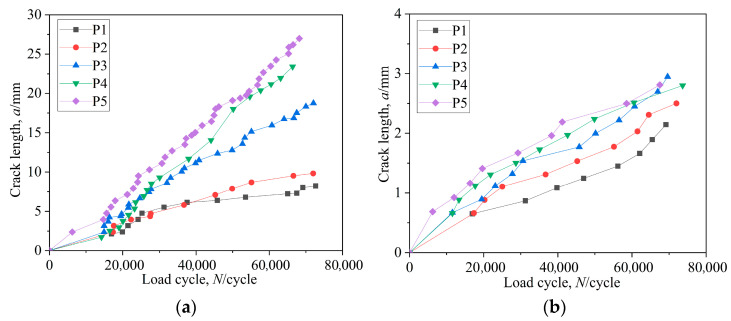
Crack propagation process in the tooth width direction and depth direction: (**a**) tooth width direction; (**b**) depth direction.

The process of crack propagation was analyzed in the tooth width direction after initiation of cracks at different positions, as illustrated in Figure 17a. When the crack initiated at the middle of root surface, i.e., position P5, the crack propagated at the maximum rate.

The process of crack propagation was analyzed in the depth direction after initiation of cracks at different positions, as illustrated in Figure 17b. In the early stage of crack propagation, there was little difference in crack propagation rate at different locations. At the later stage, the crack propagation rate increased obviously after the cracks initiated at positions P3, resulting in the depth being largest at the position. The corresponding number of cycles obtained by the test fluctuated within a certain range under the same stress conditions, as shown by existing research results. Also, the influence of the initial crack position on the fatigue life was not considered in the test process [25].

**Figure 18:**
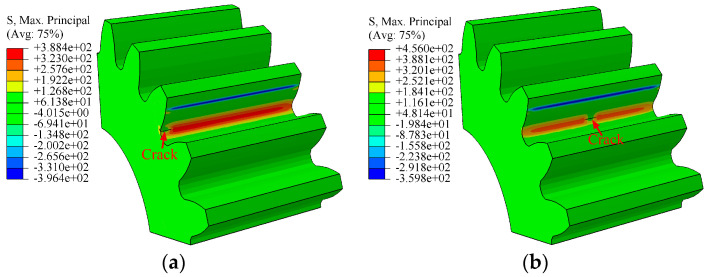
Tooth surface tensile stress: (**a**) crack initiation at position P1; (**b**) crack initiation at position P5.

After the crack initiated at positions P1 and P5, there was an obvious difference in the rate of crack propagation. Cracks initiate near the end face, and under the tensile stress on the tooth surface, the crack extends in only one direction on the tooth surface, as illustrated in Figure 18a. When cracks initiate in the middle of the tooth root, the cracks extend in both positive and negative directions of the tooth width, as illustrated in Figure 18b, making the growth rate faster than that of position P1. In order to improve the service life of the gear, it was necessary to increase the strength of the middle part of the tooth root along the tooth width direction as much as possible, to avoid the appearance of initial cracks in the middle area.

**Figure 19:**
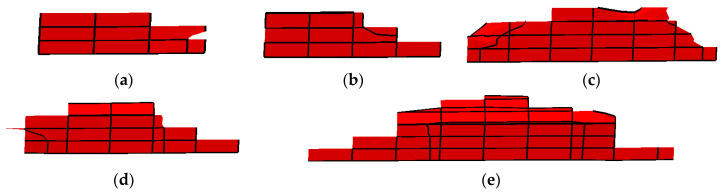
The morphology of the fracture surface after about 20,000 cycles of cyclic loading: (**a**–**e**) were the simulation results of initial cracks located at positions P1–P5, respectively.

In order to comprehensively describe the propagation process of crack in width and depth, the growth process of fracture surface was analyzed. After about 20,000 cycles of cyclic loading, the shape of the fracture surface at each position was similar and close to trapezoid, but the area of the fracture surface at P5 was significantly larger, as illustrated in Figure 19.

**Figure 20:**
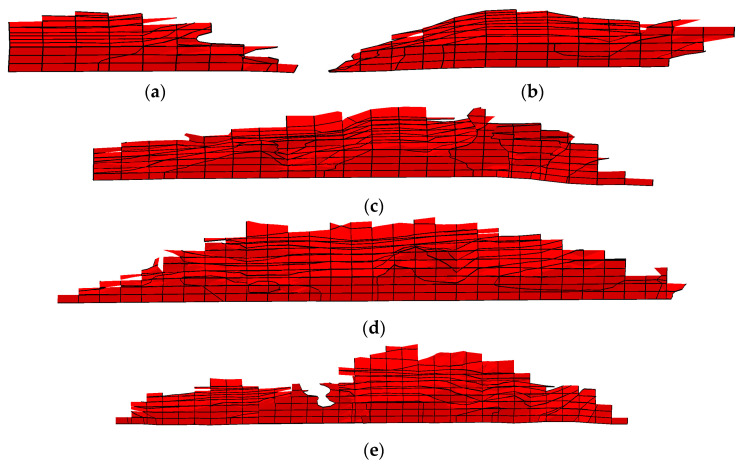
The morphology of the fracture surface after about 60,000 cycles of cyclic loading: (**a**–**e**) were the simulation results of initial cracks located at P1–P5, respectively.

**Figure 21:**
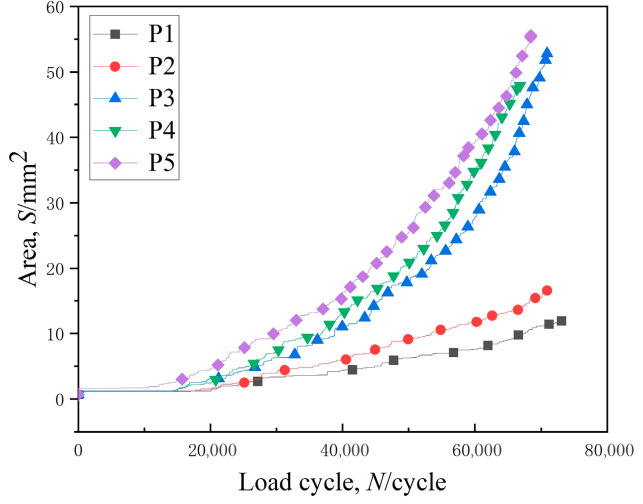
Growth of fracture area after initiation of cracks at different positions at tooth root.

After about 60,000 cycles under cyclic loading, the shape of the fracture surface was changed obviously, as illustrated in Figure 20. When the crack initiated close to the end surface of the gear, the crack was not easy to expand. The evolution process of fracture area was different for the different inceptive crack location, as shown in Figure 21. Specifically, when the crack initiated at the middle of the root surface, it propagated at a significantly faster rate, resulting in the largest fracture area.

During the inceptive phase of crack spread, the crack depth at the middle of the root surface is greater than the crack depth at the terminal face. As the crack progressively extends, the difference in crack length in the tooth width direction becomes larger, as well as the difference in crack depth. The stress strength factor at the crack front is related to the crack depth [26]. The larger the crack depth, the greater the stress intensity factor at the crack front [27,28], resulting in a faster growth rate of the whole fracture.

## 4. Conclusions

The crack propagation model of a spur gear was established by using XFEM in ABAQUS. The single-tooth bending fatigue experiment was conducted to verify the model accuracy according to the testing results of strain and fractography.

The fatigue fracture of gear teeth consisted of crack initiation and propagation under cyclic loading. The particle in tooth surface with high carbon content was the main factor for crack initiation, resulting in the appearance of initial cracks. The crack propagation process was mainly affected by the initial crack position for the certain loads and material properties.

Under the same cycle times and load conditions of the length and width area, the tooth breakage time of P3 was approximately 800 s, and the remaining life was about 80,000 times. The remaining lifetimes of P1 and P2 were greater than 100,000 times, while the remaining lifetimes of P4 and P5 ranged between 80,000 and 100,000 times.

The expansion rate of crack size in both tooth width direction and depth direction was faster as the initial crack position approached the middle of root surface. When the initial crack was located at the end surface, the change of crack area was the slowest. More cycles and fatigue life were obtained as the crack initiation position occurred on the tooth surface closer to the end surface.

In future research work, the working conditions of the gear in the actual gearbox will be considered. The calculation model in line with actual engineering applications will be established to analyze the influence of gear accuracy and installation errors on gear load, and service life will be predicted more accurately.

## Figures and Tables

**Table 1:** The gear and material parameters.

Parameter	Value
Number of teeth	23
Normal modulus/mm	4
Pressure angle/°	20
Gear width/mm	34
Material	20Cr2Ni4A
Poison ratio	0.3
Elastic modulus/GPa	206
C_1_	0.001
C_2_	0
C_3_	1.24 × 10^−4^
C_4_	1.03

**Table 2:** Coordinates of initial cracks at different locations.

Crack Location	P1	P2	P3	P4	P5
Distance from the end face/mm	0	4.2	8.3	12.4	16.5

**Table 3:** Chemical composition of 20Cr2Ni4A steel.

C	Cr	Ni	Mn	Si	Fe
0.15~0.19	1.25~1.65	3.25~3.65	0.30~0.60	0.17~0.37	Bal.

**Table 4:** The positions of strain gauges configuration.

Position of Strain Gauge	Position 1	Position 2	Position 3	Position 4
a/mm	9.1	6.56	7.0	9.1
b/mm	0	8.5	24	34

**Table 5:** Cyclic load parameters of the experiment.

Maximum Load/kN	Mean Load/kN	Alternating Load/kN	Loading Frequency/Hz	Stress Ratio	Maximum Root Bending Stress /MPa
40	20.8	19.2	100	0.04	639

**Table 6:** The position of the path in the depth direction.

Initial Crack Location	P1	P2	P3	P4	P5
Distance from the end face/mm	2.8	4.0	12.1	15.3	17.1

## Data Availability

The data presented in this study are available upon request from the corresponding author.
